# Supplementary material for: Atlas of voluntary facial muscle activation: Visualization of surface electromyographic activities of facial muscles during mimic exercises
Source: PLoS One. 2021 Jul 19;16(7):e0254932. doi: 10.1371/journal.pone.0254932 (PMC8289121; doi:10.1371/journal.pone.0254932)
Supplement: S1 Table — (DOCX) [file pone.0254932.s001.docx]

**Supplemental Table S1**

| **Supplement Table S1.** Mimic tasks in comparison to action units (AU) of the FACS system. | | | | | |
| --- | --- | --- | --- | --- | --- |
| **No.** | **Description of the task (T)** | **Muscles with highest sEMG activity** | **AU equivalent** | **AU muscular basis** |  |
| 1 | Pronouncing the German vowel: A/aː/* | inferior orbicularis oris, mentalis, depressor labii | -- | -- |  |
| 2 | Pronouncing the German vowel: Ä/æ/* | inferior orbicularis oris, depressor labii, mentalis | -- | -- |  |
| 3 | Pronouncing the German vowel: E/eː/* | inferior orbicularis oris, depressor labii, mentalis | -- | -- |  |
| 4 | Pronouncing the German vowel: I/iː/* | inferior orbicularis oris, depressor labii, mentalis | -- | -- |  |
| 5 | Pronouncing the German vowel: O/oː/* | inferior orbicularis oris, depressor labii | -- | -- |  |
| 6 | Pronouncing the German vowel: U/uː/* | inferior orbicularis oris, depressor labii | -- | -- |  |
| 7 | Pressing lips together | orbicularis oris, depressor anguli oris, mentalis, depressor labii, zygomatic | AU 23 Lip tightener | orbicularis oris |  |
| 8 | Pulling corners of the mouth downwards | inferior orbicularis oris, depressor anguli oris, depressor labii, mentalis | AU 15 Lip corner depressor | depressor anguli oris |  |
| 9 | Voluntary smiling: Pulling corners of the mouth upwards and backwards | zygomatic, orbicularis oris, mentalis, depressor labii, depressor anguli oris | -- | -- |  |
| 10 | Depressing lower lip | inferior orbicularis oris, depressor labii, mentalis | AU 16 Lower lip depressor | depressor labii |  |
| 11 | Protruding lower lip | mentalis, depressor anguli oris, depressor labii, orbicularis oris | -- | -- |  |
| 12 | Pulling upper lip upwards | levator labii superioris alaeque nasi, levator labii superioris, orbicularis oris | AU 10 Upper lip raiser | levator labii superioris |  |
| 13 | Pulling upper lip upwards and depressing lower lip simultaneously | depressor labii, inferior orbicularis oris, mentalis | AU 20 Lip stretcher | risorius |  |
| 14 | Pursing lips | orbicularis oris | AU 18 Lip puckerer | orbicularis oris |  |
| 15 | Blowing out cheeks | orbicularis oris, mentalis, depressor labii, | -- | -- |  |
| 16 | Sucking cheeks inward | orbicularis oris, mentalis, depressor labii, | -- | -- |  |
| 17 | Whistling with a similar tone pitch | orbicularis oris | -- | -- |  |
| 18 | Opening jaw with closed lips | mentalis, orbicularis oris, depressor labii, depressor anguli oris | -- | -- |  |
| 19 | Exhaling forcefully with moderate closed lips | orbicularis oris | -- | -- |  |
| 20 | Opening lips as wide as possible while the jaw is closed | inferior orbicularis oris, depressor labii, mentalis | AU 27 Mouth stretch | pterygoid, digastric |  |
| 21 | Wrinkling the nose | levator labii superioris alaeque nasi | AU 9 Nose wrinkle | levator labii superioris alaeque nasi |  |
| 22 | Voluntary smiling only on right side of the face | zygomatic | AU 12 Lip corner puller | zygomaticus major |  |
| 23 | Voluntary smiling only on left side of the face | zygomatic | AU 12 Lip corner puller | zygomaticus major |  |
| 24 | Raising eyebrows up and wrinkling the forehead | frontalis | AU 1/2 Brow raiser | frontalis |  |
| 25 | Contracting eyebrows | frontalis | AU 4 Brow lowerer | depressor, corrugator supercilii |  |
| 26 | Closing eyelids forcefully | orbicularis oculi, frontalis | AU 43 | orbicularis oculi |  |
| 27 | Squinting the eyes | orbicularis oculi, frontalis | AU 45/AU 46 | orbicularis oculi |  |
| 28 | Closing the right eyelid | right orbicularis oculi, right frontalis, right levator labii superioris | AU 43 | orbicularis oculi |  |
| 29 | Closing the left eyelid | left orbicularis oculi, left frontalis, left levator labii superioris | AU 43 | orbicularis oculi |  |
